# Supplementary material for: 4-(Phenylselanyl)-2H-chromen-2-one-Loaded Nanocapsule Suspension—A Promising Breakthrough in Pain Management: Comprehensive Molecular Docking, Formulation Design, and Toxicological and Pharmacological Assessments in Mice
Source: Pharmaceutics. 2024 Feb 14;16(2):269. doi: 10.3390/pharmaceutics16020269 (PMC10892109; doi:10.3390/pharmaceutics16020269)
Supplement: Supplementary file 1 [file pharmaceutics-16-00269-s001.zip › pharmaceutics-2840976-supplementary.pdf]

Supplementary Material

4-(Phenylselanyl)-2H-chromen-2-one-loaded Nanocapsule Suspension - A Promising Breakthrough in Pain Management: Comprehensive Molecular Docking, Formulation Design, Toxicological, and Pharmacological Assessments in Mice

Caren Aline Ramson da Fonseca[1], Vinicius Costa Prado[2], Jaini Janke Paltian[1], Jean Carlo Kazmierczak[3], Ricardo Frederico Schumacher[3], Marcel Henrique Marcondes Sari[2], Larissa Marafiga Cordeiro[4], Aline Franzen da Silva[4], Felix Alexandre Antunes Soares[4], Robson da Silva Oliboni[5], Cristiane Luchese[1], Letícia Cruz[2*] and Ethel Antunes Wilhelm[1*]

1 Graduate Program in Biochemistry and Bioprospecting, Biochemical Pharmacology Research Laboratory, Federal University of Pelotas, Pelotas, CEP 96010-900, RS, Brazil; carenramson@hotmail.com; jaini_paltian@hotmail.com; cristiane_luchese@yahoo.com.br; *Correspondence: ethelwilhelm@yahoo.com.br

2 Graduate Program in Pharmaceutical Sciences, Pharmaceutical Technology Laboratory, Federal University of Santa Maria, Santa Maria, CEP 97105-900, RS, Brazil; vini132007@gmail.com; marcelsarih@hotmail.com; *Correspondence: leticia.cruz@ufsm.br

3 Graduate Program in Chemistry, Chemistry Department, Federal University of Santa Maria, Santa Maria, CEP 97105-900, RS, Brazil; jeanckazmierczak@gmail.com; ricardo.schumacher@ufsm.br

4 Graduate Program in Biological Sciences: Toxicological Biochemistry, Federal University of Santa Maria, Santa Maria, CEP 97105-900, RS, Brazil; larissa.marafiga@hotmail.com; alinefranzen@hotmail.com; felix@ufsm.br

5 Center for Chemical, Pharmaceutical, and Food Sciences, CCQFA, Federal University of Pelotas, Pelotas, CEP 96010-900, RS, Brazil; rooliboni@gmail.com

SUPPLEMENTARY FIGURES

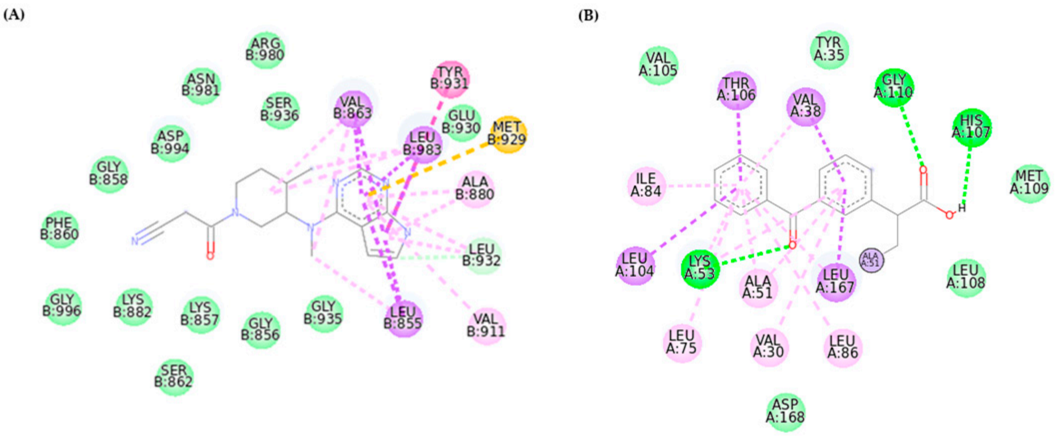

Figure S1. Interaction maps for (A) tofacitinib with JAK and (B) ketoprofen with MAPK.

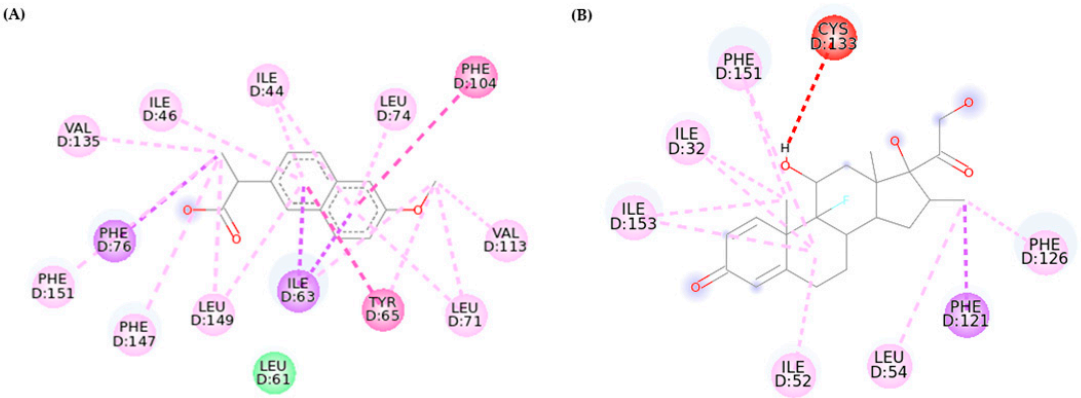

Figure S2. Interaction maps for (A) naproxen and (B) dexamethasone with TLR4.

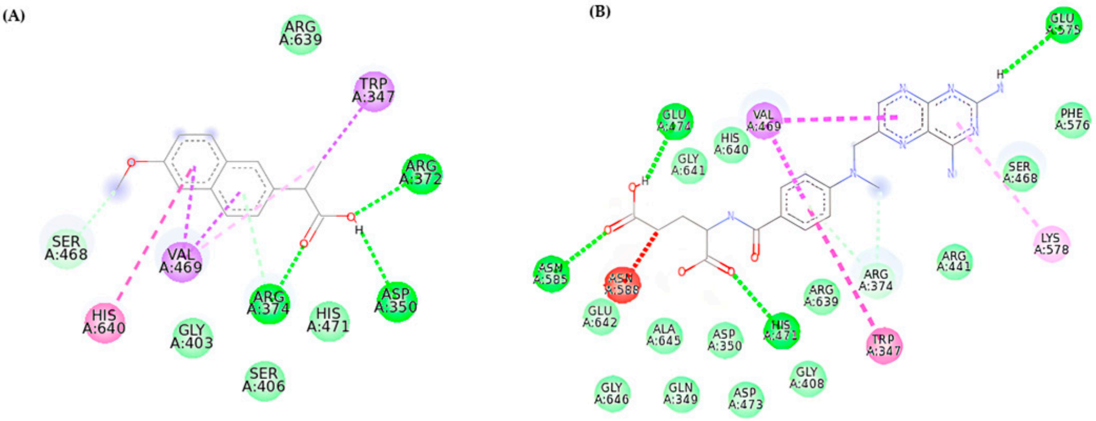

Figure S3. Interaction maps for (A) naproxen and (B) methotrexate with PAD4.

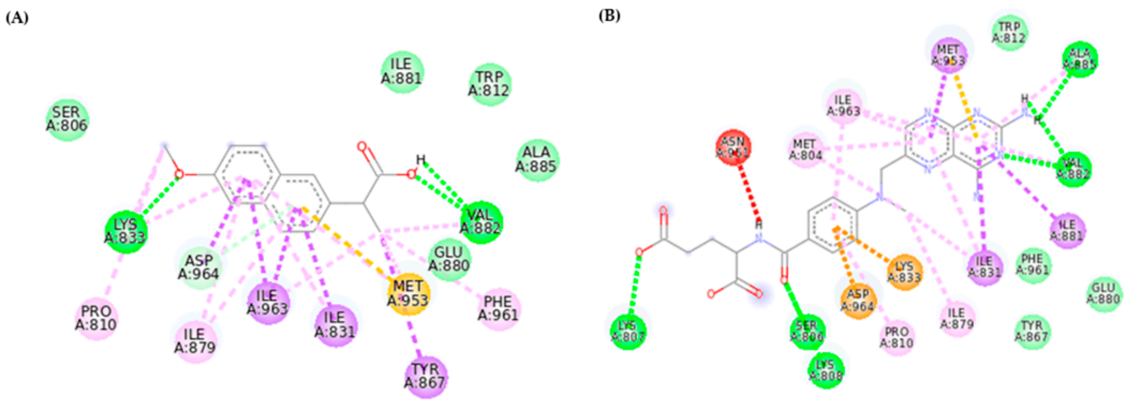

Figure S4. Interaction maps for (A) naproxen and (B) methotrexate with PI3K.

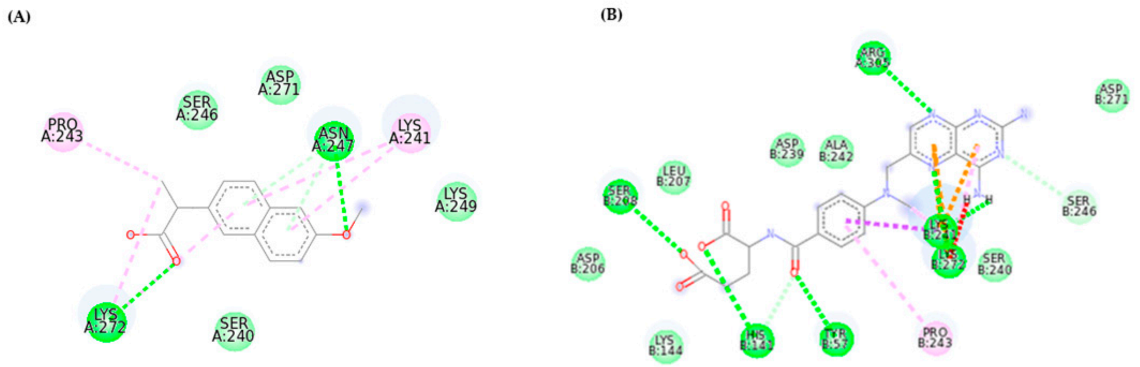

Figure S5. Interaction maps for (A) naproxen and (B) methotrexate with NFkB.

(A)

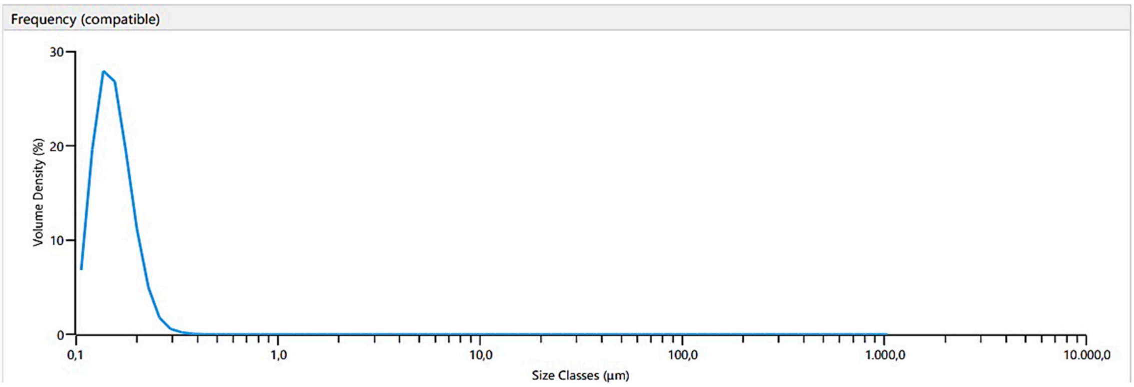

(B)

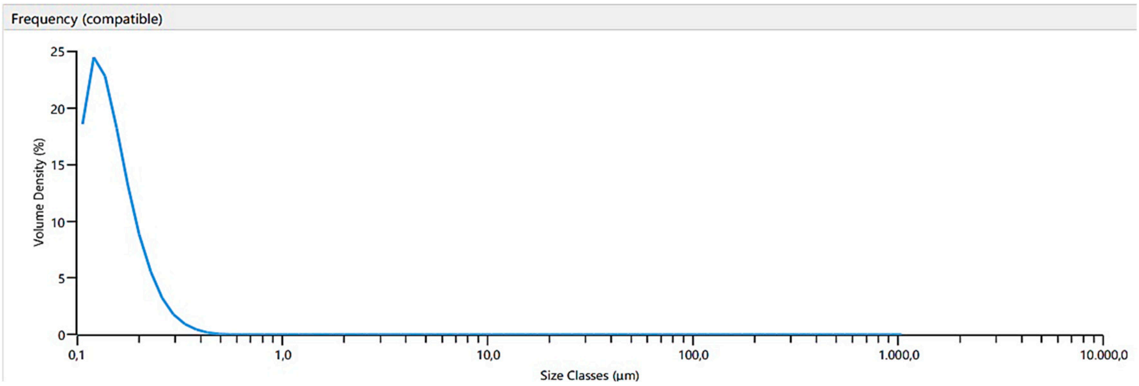

Figure S6: Representative granulometric profiles determined by laser diffraction at their initial time of 4-PSCO NC (**Figure S6A**) and NC P (**Figure S6B**), respectively. The nanoparticles population is in the nanometric range (< 1 μm).

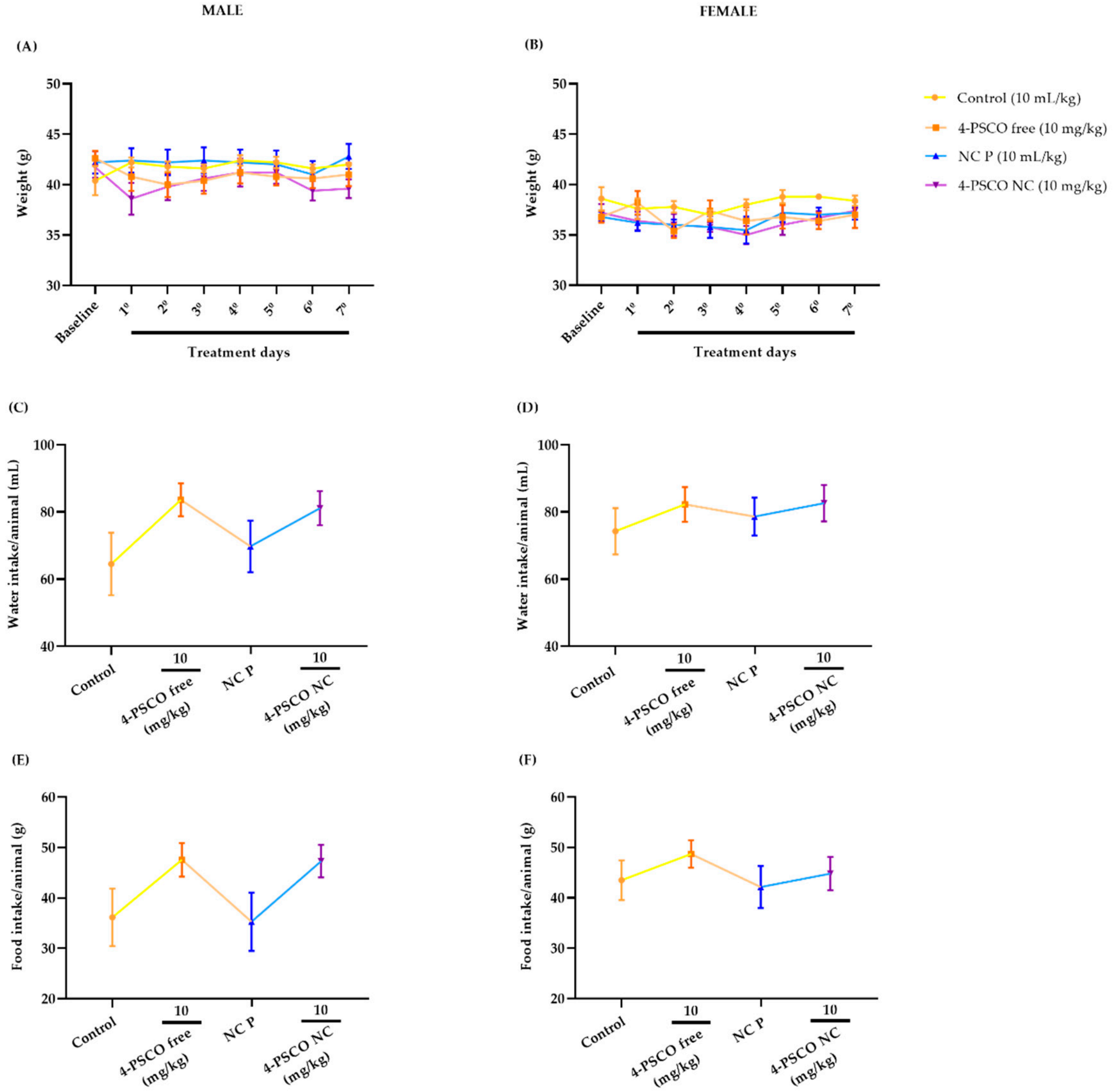

Figure S7: Effect of 4-PSCO free (10 mg/kg), 4-PSCO NC (10 mg/kg), and NC P (10 mL/kg) repeated treatment in body weight of male **(Figure S7A)** and female **(Figure S7B)** animals, water intake of male **(Figure S7C)** and female **(Figure S7D)** mice and food intake of male **(Figure S7E)** and female **(Figure S7F)** mice during the experiment. Each column represents the mean ± S.E.M. of 5 mice in each group. Data were analyzed by one-way ANOVA followed by the Tukey's test ($p > 0.05$).

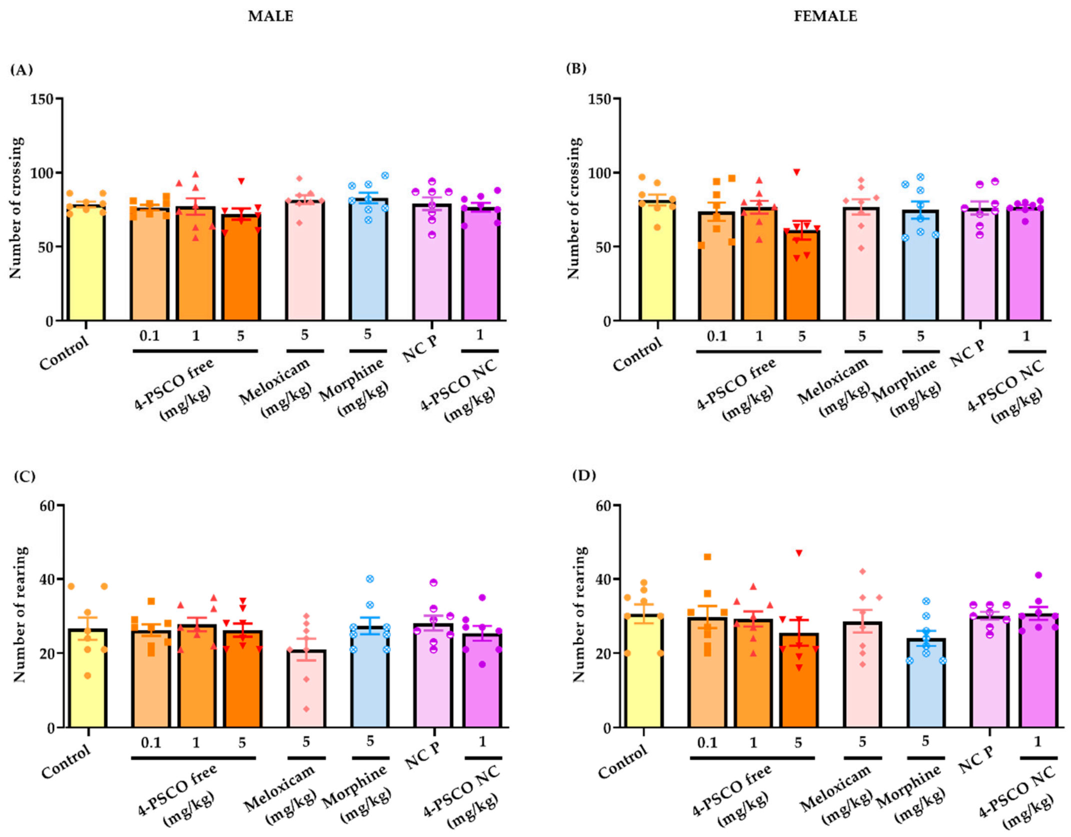

Figure S8: Effect of 4-PSCO free (0.1, 1, and 5 mg/kg), 4-PSCO NC (1 mg/kg), NC P (10 mL/kg), meloxicam (5 mg/kg), and morphine (5 mg/kg) on open field test - number of crossing **(Figures S8A and S8B),** and number of rearing **(Figures S8C and S8D)** in male and female mice. Each column represents the mean ± S.E.M. of 8 mice in each group (one-way ANOVA followed by the Tukey's test) ($p > 0.05$).

SUPPLEMENTARY TABLES

Table S1: The absolute and relative organ weight of mice treated orally with 4-PSCO free, NC P, and 4-PSCO NC in a short-term toxicity study.

	Tissues		
	**SPLEEN**	**LIVER**	**KIDNEYS**
**Experimental groups**		**Parameter**	
**MALE**		**Absolute organ weight (g)**	
**Control (10 mL/kg)**	0.13 ± 0.01	0.58 ± 0.04	1.93 ± 0.05
**4-PSCO free (10 mg/kg)**	0.11 ± 0.01	0.59 ± 0.01	2.00 ± 0.15
**NC P (10 mL/kg)**	0.12 ± 0.01	0.61 ± 0.03	2.1 ± 0.06
**4-PSCO NC (10 mg/kg)**	0.10 ± 0.01	0.56 ± 0.01	2.1 ± 0.10
		**Relative organ weight (%)**	
**Control (10 mL/kg)**	0.31 ± 0.02	4.61 ± 0.13	1.40 ± 0.11
**4-PSCO free (10 mg/kg)**	0.26 ± 0.03	4.84 ± 0.23	1.44 ± 0.06
**NC P (10 mL/kg)**	0.28 ± 0.01	4.82 ± 0.18	1.41 ± 0.05
**4-PSCO NC (10 mg/kg)**	0.25 ± 0.02	5.32 ± 0.22	1.42 ± 0.06
**FEMALE**		**Absolute organ weight (g)**	
**Control (10 mL/kg)**	0.15 ± 0.01	0.43 ± 0.02	1.81 ± 0.05
**4-PSCO free (10 mg/kg)**	0.14 ± 0.01	0.41 ± 0.02	1.81 ± 0.15
**NC P (10 mL/kg)**	0.13 ± 0.01	0.44 ± 0.01	1.82 ± 0.17
**4-PSCO NC (10 mg/kg)**	0.12 ± 0.01	0.42 ± 0.02	1.7 ± 0.15
		**Relative organ weight (%)**	
**Control (10 mL/kg)**	0.39 ± 0.02	4.71 ± 0.12	1.12 ± 0.06
**4-PSCO free (10 mg/kg)**	0.38 ± 0.03	4.91 ± 0.40	1.09 ± 0.04
**NC P (10 mL/kg)**	0.35 ± 0.01	4.88 ± 0.44	1.19 ± 0.02
**4-PSCO NC (10 mg/kg)**	0.33 ± 0.01	4.54 ± 0.39	1.13 ± 0.04

The values are reported as mean ± S.E.M. The data were analyzed using a one-way ANOVA followed by Tukey multiple comparison test. The relative weight (g) was calculated as (organ weight (g)/body weight of the animal on sacrifice day (g)) × 100).

Table S2: Results of statistical analyses of the water consumption, food consumption, and body weight of mice treated orally with 4-PSCO free, NC P, and 4-PSCO NC in a short-term toxicity study.

Parameter	MALE	FEMALE
**Water consumption**	$F_{(3, 28)} = 1.703$, $p > 0.05$	$F_{(3, 28)} = 0.4482$, $p > 0.05$
**Food consumption**	$F_{(3, 28)} = 2.104$, $p > 0.05$	$F_{(3, 28)} = 0.6245$, $p > 0.05$
**Body weight (Baseline)**	$F_{(3, 16)} = 0.6402$, $p > 0.05$	$F_{(3, 16)} = 1.090$, $p > 0.05$
**Body weight (Day 1)**	$F_{(3, 16)} = 1.508$, $p > 0.05$	$F_{(3, 16)} = 0.9436$, $p > 0.05$
**Body weight (Day 2)**	$F_{(3, 16)} = 1.174$, $p > 0.05$	$F_{(3, 16)} = 1.878$, $p > 0.05$
**Body weight (Day 3)**	$F_{(3, 16)} = 0.6991$, $p > 0.05$	$F_{(3, 16)} = 1.030$, $p > 0.05$
**Body weight (Day 4)**	$F_{(3, 16)} = 0.3293$, $p > 0.05$	$F_{(3, 16)} = 1.464$, $p > 0.05$
**Body weight (Day 5)**	$F_{(3, 16)} = 0.4139$, $p > 0.05$	$F_{(3, 16)} = 1.532$, $p > 0.05$
**Body weight (Day 6)**	$F_{(3, 16)} = 0.9136$, $p > 0.05$	$F_{(3, 16)} = 2.846$, $p > 0.05$
**Body weight (Day 7)**	$F_{(3, 16)} = 2.058$, $p > 0.05$	$F_{(3, 16)} = 0.6042$, $p > 0.05$

The data were analyzed using a one-way ANOVA followed by Tukey multiple comparison test.

Table S3: Results of statistical analyses of the absolute organ weight of mice treated orally with 4-PSCO free, NC P, and 4-PSCO NC in a short-term toxicity study.

**Absolute organ weight (g)**	
**MALE**	**FEMALE**
**SPLEEN**	**SPLEEN**
$F_{(3, 16)} = 1.739$, $p > 0.05$	$F_{(3, 16)} = 1.893$, $p > 0.05$
**LIVER**	**LIVER**
$F_{(3, 16)} = 2.381$, $p > 0.05$	$F_{(3, 16)} = 0.2263$, $p > 0.05$
**KIDNEYS**	**KIDNEYS**
$F_{(3, 16)} = 0.09429$, $p > 0.05$	$F_{(3, 16)} = 0.8628$, $p > 0.05$

Data were evaluated through ordinary one-way ANOVA followed by the Tukey's test ($p > 0.05$).

Table S4: Thiobarbituric acid reactive species (TBARS) levels of male and female mice submitted to the repeated treatment with 4-PSCO free or 4-PSCO NC.

	**Tissues**			
	**BRAIN**	**SPINAL CORD**	**LIVER**	**KIDNEYS**
**Experimental groups**	**Parameter**			
**MALE**	**TBARS (nmol MDA/mg protein)**			
**Control (10 mL/kg)**	18.8 ± 1.0	20.4 ± 1.4	61.7 ± 2.8	29.5 ± 2.3
**4-PSCO free (10 mg/kg)**	18.5 ± 1.3	17.2 ± 1.2	66.5 ± 4.8	35.6 ± 2.7
**NC P (10 mL/kg)**	22.0 ± 2.1	19.7 ± 1.4	66.9 ± 4.5	34.6 ± 4.6
**4-PSCO NC (10 mg/kg)**	17.3 ± 0.5	19.6 ± 0.9	65.0 ± 1.7	31.8 ± 2.4
**FEMALE**	**TBARS (nmol MDA/mg protein)**			
**Control (10 mL/kg)**	20.9 ± 1.5	24.4 ± 1.1	72.2 ± 3.1	31.5 ± 1.5
**4-PSCO free (10 mg/kg)**	22.8 ± 1.5	22.8 ± 1.4	73.2 ± 2.5	32.9 ± 3.0
**NC P (10 mL/kg)**	19.1 ± 2.0	20.9 ± 0.7	69.0 ± 2.7	36.3 ± 3.6
**4-PSCO NC (10 mg/kg)**	21.7 ± 1.9	22.8 ± 2.1	77.7 ± 1.7	35.2 ± 2.0

Values are expressed as mean ± S.E.M. of 5 animals/group to each parameter. Data were evaluated through ordinary one-way ANOVA followed by the Tukey's test ($p > 0.05$).

Table S5: Non-protein thiol (NPSH) levels of male and female mice submitted to the repeated treatment with 4-PSCO free or 4-PSCO NC.

	Tissues			
	**BRAIN**	**SPINAL CORD**	**LIVER**	**KIDNEYS**
**Experimental groups**	**Parameter**			
**MALE**	**NPSH (nmol of NPSH/g tissue)**			
**Control (10 mL/kg)**	2.7 ± 0.2	3.3 ± 0.1	3.5 ± 0.2	3.2 ± 0.1
**4-PSCO free (10 mg/kg)**	2.4 ± 0.1	3.7 ± 0.1	3.4 ± 0.1	3.2 ± 0.1
**NC P (10 mL/kg)**	2.5 ± 0.3	3.4 ± 0.1	3.4 ± 0.2	3.6 ± 0.3
**4-PSCO NC (10 mg/kg)**	3.1 ± 0.1	3.6 ± 0.2	3.7 ± 0.2	3.2 ± 0.1
**FEMALE**	**NPSH (nmol of NPSH/g tissue)**			
**Control (10 mL/kg)**	3.2 ± 0.1	3.6 ± 0.2	3.4 ± 0.1	3.2 ± 0.1
**4-PSCO free (10 mg/kg)**	3.0 ± 0.1	3.8 ± 0.3	3.1 ± 0.1	3.3 ± 0.1
**NC P (10 mL/kg)**	2.9 ± 0.1	4.1 ± 0.3	3.1 ± 0.1	3.1 ± 0.1
**4-PSCO NC (10 mg/kg)**	2.8 ± 0.3	4.1 ± 0.2	3.2 ± 0.2	3.2 ± 0.2

Values are expressed as mean ± S.E.M. of 5 animals/group to each parameter. Data were evaluated through ordinary one-way ANOVA followed by the Tukey's test ($p > 0.05$).

Table S6: Results of statistical analyses of thiobarbituric acid reactive species (TBARS) and non-protein thiol (NPSH) levels of male and female mice subjected to repeated treatment with 4-PSCO free or 4-PSCO NC.

TBARS (nmol MDA/mg protein)	
**MALE**	**FEMALE**
**BRAIN**	**BRAIN**
$F_{(3, 16)} = 2.121$, $p > 0.05$	$F_{(3, 16)} = 0.7890$, $p > 0.05$
**SPINAL CORD**	**SPINAL CORD**
$F_{(3, 16)} = 1.286$, $p > 0.05$	$F_{(3, 16)} = 0.9531$, $p > 0.05$
**LIVER**	**LIVER**
$F_{(3, 16)} = 0.4185$, $p > 0.05$	$F_{(3, 16)} = 2.021$, $p > 0.05$
**KIDNEYS**	**KIDNEYS**
$F_{(3, 16)} = 0.7757$, $p > 0.05$	$F_{(3, 16)} = 0.6706$, $p > 0.05$
**NPSH (nmol of NPSH/g tissue)**	
**BRAIN**	**BRAIN**
$F_{(3, 16)} = 3.172$, $p > 0.05$	$F_{(3, 16)} = 0.7593$, $p > 0.05$
**SPINAL CORD**	**SPINAL CORD**
$F_{(3, 16)} = 0.8110$, $p > 0.05$	$F_{(3, 16)} = 0.9031$, $p > 0.05$
**LIVER**	**LIVER**
$F_{(3, 16)} = 0.8830$, $p > 0.05$	$F_{(3, 16)} = 0.8644$, $p > 0.05$
**KIDNEYS**	**KIDNEYS**
$F_{(3, 16)} = 1.342$, $p > 0.05$	$F_{(3, 16)} = 0.1715$, $p > 0.05$

Data were evaluated through ordinary one-way ANOVA followed by the Tukey's test ($p > 0.05$).

Table S7: Alanine aminotransferase (ALT), aspartate aminotransferase (AST) activities, and urea levels of male and female mice submitted to the repeated treatment with 4-PSCO free or 4-PSCO NC.

Tissues			
**PLASMA**			
**Parameter**			
**MALE**	**ALT (U/L)**	**AST (U/L)**	**UREA (mg/dL)**
**Control (10 mL/kg)**	8.1 ± 1.7	29.7 ± 4.5	29.0 ± 1.7
**4-PSCO free (10 mg/kg)**	11.4 ± 1.8	27.8 ± 5.9	23.3 ± 1.7
**NC P (10 mL/kg)**	10.1 ± 0.7	19.6 ± 2.7	31.1 ± 1.6
**4-PSCO NC (10 mg/kg)**	8.7 ± 0.6	25.1 ± 7.4	28.0 ± 1.9
**FEMALE**	**ALT (U/L)**	**AST (U/L)**	**UREA (mg/dL)**
**Control (10 mL/kg)**	10.2 ± 1.9	17.1 ± 2.5	30.8 ± 2.4
**4-PSCO free (10 mg/kg)**	10.7 ± 3.1	18.7 ± 2.2	24.6 ± 1.6
**NC P (10 mL/kg)**	12.7 ± 1.6	15.2 ± 3.0	26.2 ± 1.7
**4-PSCO NC (10 mg/kg)**	10.3 ± 1.5	16.0 ± 1.7	26.6 ± 1.5

Values are expressed as mean ± S.E.M. of 5 animals/group to each parameter. Data were evaluated through ordinary one-way ANOVA followed by the Tukey's test ($p > 0.05$).

Table S8: Results of statistical analyses of alanine aminotransferase (ALT), aspartate aminotransferase (AST), and Urea levels in male and female mice subjected to repeated treatment with 4-PSCO free or 4-PSCO NC.

ALT (U/L)	
**MALE**	**FEMALE**
**PLASMA**	**PLASMA**
$F_{(3, 16)} = 1.291$, $p > 0.05$	$F_{(3, 16)} = 0.2982$, $p > 0.05$
**AST (U/L)**	
$F_{(3, 16)} = 0.6416$, $p > 0.05$	$F_{(3, 16)} = 0.3836$, $p > 0.05$
**UREA (mg/dL)**	
$F_{(3, 16)} = 3.646$ $p > 0.05$	$F_{(3, 16)} = 2.000$, $p > 0.05$

Data were evaluated through ordinary one-way ANOVA followed by the Tukey's test ($p > 0.05$).
